# Synergistic Effect of Mesangial Cell-Induced CXCL1 and TGF-β1 in Promoting Podocyte Loss in IgA Nephropathy

**DOI:** 10.1371/journal.pone.0073425

**Published:** 2013-08-30

**Authors:** Li Zhu, Qingxian Zhang, Sufang Shi, Lijun Liu, Jicheng Lv, Hong Zhang

**Affiliations:** 1 Renal Division, Department of Medicine, Peking University First Hospital, Beijing, China; 2 Peking University Institute of Nephrology, Beijing, China; 3 Key Laboratory of Renal Disease, Ministry of Health of China, Beijing, China; 4 Key Laboratory of Chronic Kidney Disease Prevention and Treatment (Peking University), Ministry of Education, Beijing, China; INSERM, France

## Abstract

Podocyte loss has been reported to relate to disease severity and progression in IgA nephropathy (IgAN). However, the underlying mechanism for its role in IgAN remain unclear. Recent evidence has shown that IgA1 complexes from patients with IgAN could activate mesangial cells to induce soluble mediator excretion, and further injure podocytes through mesangial-podocytic cross-talk. In the present study, we explored the underlying mechanism of mesangial cell-induced podocyte loss in IgAN. We found that IgA1 complexes from IgAN patients significantly up-regulated the expression of CXCL1 and TGF-β1 in mesangial cells compared with healthy controls. Significantly higher urinary levels of CXCL1 and TGF-β1 were also observed in patients with IgAN compared to healthy controls. Moreover, IgAN patients with higher urinary CXCL1 and TGF-β1 presented with severe clinical and pathological manifestations, including higher 24-hour urine protein excretion, lower eGFR and higher cresentic glomeruli proportion. Further in vitro experiments showed that increased podocyte death and reduced podocyte adhesion were induced by mesangial cell conditional medium from IgAN (IgAN-HMCM), as well as rhCXCL1 together with rhTGF-β1. In addition, the over-expression of CXCR2, the receptor for CXCL1, by podocytes was induced by IgAN-HMCM and rhTGF-β1, but not by rhCXCL1. Furthermore, the effect of increased podocyte death and reduced podocyte adhesion induced by IgAN-HMCM and rhCXCL1 and rhTGF-β1 was rescued partially by a blocking antibody against CXCR2. Moreover, we observed the expression of CXCR2 in urine exfoliated podocytes in IgAN patients. Our present study implied that IgA1 complexes from IgAN patients could up-regulate the secretion of CXCL1 and TGF-β1 in mesangial cells. Additionally, the synergistic effect of CXCL1 and TGF-β1 further induced podocyte death and adhesion dysfunction in podocytes via CXCR2. This might be a potential mechanism for podocyte loss observed in IgAN.

## Introduction

Immunoglobulin A nephropathy (IgAN) is the most common form of glomerulonephritis worldwide and one of the main causes of end stage renal disease (ESRD) [1,2]. Although the precise pathogenesis is still undetermined, the basic abnormality of IgAN is considered within the IgA immune system rather than in the kidney [3]. Reports have shown the recurrence of IgA deposits after transplantation in IgAN patients, as well as the reversion of IgA deposits after transplantation in patients who were inadvertently grafted kidneys with IgA deposits [4–6]. In recent years, emerging evidence has indicated the key role of circulating complexes that contain aberrant glycosylated IgA1 in driving their mesangial deposition and the triggering of glomerular injury in IgAN [7–11], even if the exact composition and physicochemical characteristics of the circulating complex remain unclear.

The diagnostic histological features of IgAN are the deposition of pathogenic IgA1 complexes (cIgA1) in the glomerular mesangium [1,2], which indicates mesangial cell as the first target of injury. In addition to this, injury to other renal intrinsic cells is also observed in a considerable proportion of patients with IgAN. Several studies reported podocyte loss in the renal tissues of IgAN patients and further identified the predictive role of podocytopenia in IgAN progression [12–14]. Podocytes are positioned along the glomerular basement membrane to form the outer exocapillary layer of the glomerular filtration barrier. Previous animal studies have proved that podocyte loss could lead to proteinuria and glomerulosclerosis [15], which are features usually observed in patients with IgAN. However, the mechanism of podocyte loss in IgAN is not clear.

Recently, *in vitro* studies have found that podocytes treated with conditioned medium from mesangial cells preincubated with pathogenic IgA1 can induce nephrin loss and cytoskeleton rearrangement; this action is mediated by humoral factors released from mesangial cells, predominantly TGF-β1 and TNF-α [8,9,16]. Based on these podocyte culture experiments, mesangial-podocytic cross-talk has been postulated as a new mechanism in the pathogenesis of IgAN [7].

As IgA is rarely deposited in podocytes themselves [9], it is a likely possibility that mesangial IgA1 deposition is a kind of danger signal; accordingly, mesangial cells show several danger responses, one of which is multiple cytokines release; the released cytokines may lead to a maladaptive process inside the glomerulus, such as podocyte loss [17]. In the present study, using IgA1 complexes derived from patients with IgAN, we investigated whether pathologic mediators excreted by mesangial cells could promote podocyte loss in IgAN.

## Materials and Methods

### Ethics Statement

The study protocol was reviewed and approved by the Ethics Committee of Peking University and written informed consent was obtained from all participants.

### Patients and controls

In the present study, 151 renal-biopsy proven primary IgAN patients were enrolled. Among them, 28 patients (17 male and 11 female) were used for plasma IgA1 purification, 104 patients (55 male and 49 female) were enrolled for urinary cytokine examinations, and another 19 patients participated in the examination of urinary podocytes. The diagnoses of IgAN were confirmed by the observation of granular IgA deposition in the glomerular mesangium by an immunofluorescent detection method, as well as by the deposition of electron dense materials observed by mesangial ultra-structural examinations. Patients with Henoch-Schönlein purpura, systemic lupus erythematosus and chronic hepatic diseases were excluded after detailed clinical and laboratory examinations. Clinical and pathological manifestations at the time of renal biopsy, including serum creatinine levels, 24-hour urine protein excretion (UPE), mean arterial pressure (MAP) and the number of crescentic glomeruli were collected from the clinical records. The glomerular filtration rate (GFR) of IgAN patients was calculated by the Modified Glomerular Filtration Rate Estimating Equation for a Chinese Population [18]. Accordingly, 94 healthy subjects (54 male and 40 female), who were comparable in terms of age and geographical origin, with no microscopic hematuria or proteinuria, were recruited as controls (20 for IgA1 isolation and 74 for urinary cytokine examinations).

### Isolation of IgA1 complexes

On the morning of the renal biopsy (for IgAN patients) or recruitment (for healthy controls), 10 ml of blood were collected from each enrolled subject. Then, plasma was isolated and frozen at -80°C immediately pending IgA1 isolation. IgA1 was first purified from plasma by an agarose-bound jacalin affinity chromatography column (Pierce Chemical Company, Rockford, IL, USA), as previously described [19]. Briefly, the plasma was diluted 1:2 with 0.01 M phosphate buffered saline (PBS, pH 7.4), filtered through a 0.2-μm filter and then applied to the jacalin column. The column was equilibrated with 0.01 M PBS, and then IgA1 was eluted with 0.1 M melibiose (Sigma, St. Louis, MO, USA) in 0.01 M PBS. The eluted fractions were collected and concentrated by pressure ultrafiltration using Vivaspin (Sartorius Stedim Biotech, Sartorius, Goettingen, Germany). The purified IgA1 was then applied to a Sephacryl S-300 gel filtration chromatography column (GE Healthcare Life Sciences, Uppsala, Sweden). There were three peaks at 280 nm, which represented IgA1 complexes (cIgA1), monomeric IgA1 (mIgA1) and other small proteins. These fractions were pooled separately and concentrated. The purified cIgA1from IgAN patients (IgAN-cIgA1) and healthy controls (HC-cIgA1) were identified by both Western blotting and ELISA.

### Mesangial cell culture and treatment

Primary human renal mesangial cells and all the cell culture media, supplements and fetal bovine serum (FBS) were purchased from ScienCell^TM^ Corporation (ScienCell^TM^, Carlsbad, CA, USA). Cells were cultured according to the manufacturer’s specifications in mesangial cell medium (MCM) supplemented with mesangial cell growth supplement (MsCGS), 5% FBS, penicillin G (100 U/ml) and streptomycin (100 U/ml) at 37°C in a humidified 5% CO_2_ incubator. After cell growth was arrested for 16 hours without FBS, HMCs were incubated with 100 μg/ml cIgA1 isolated from the sera of IgAN patients (IgAN) or healthy controls (HC) for 48 hours. The supernatants, after centrifugation, were collected and stored at -80°C until their use in further experiments.

### Podocyte culture and treatment

We used previously established and reported conditionally immortalized human podocyte cell line (gift from Professor Moin A. Saleem, Children’s Renal Unit and Academic Renal Unit, University of Bristol, UK) in the present study [20]. Podocytes were maintained at 33°C to promote cell propagation and moved to 37°C for differentiation as previously described [20]. After being arrested overnight with 0.5% FBS, differentiated podocytes were seeded into plates (for adhesion assay, Lab-Tek^TM^ chamber slides [Nunc, Roskilde, Denmark] were used). For mesangial-podocytic communication assays, podocytes were incubated with conditioned medium from non-treated HMCs (HMCM 1:8 dilution), conditioned medium from IgAN-cIgA1-treated HMCs (IgAN-HMCM 1:8 dilution), conditioned medium from HC-cIgA1-treated HMCs (HC-HMCM 1:8 dilution), recombinant human TGF-β1 (rhTGF-β1 2 μg/ml), recombinant human CXCL1 (rhCXCL1 2 μg/ml) and combined rhTGF-β1 and rhCXCL1 (rhTGF-β1 2 μg/ml, rhCXCL1 2 μg/ml), respectively. For the inhibition assay, podocytes were also incubated with 0.5 µg/ml of a blocking antibody of CXCR2, the receptor for CXCL1.

### Cytokine array

To detect the cytokine profile released by mesangial cells, a Proteome Profiler Human Cytokine Array Kit Panel A (R&D Systems, Minneapolis, MN, USA) was used according to the manufacturer’s specifications. Briefly, 1 ml culture medium and the detection antibody cocktail were incubated with the array membrane at 4°C overnight. After washes, the array membranes were incubated with HRP-conjugated streptavidin. The blots were detected with western lightning plus ECL reagent (PerkinElmer, Waltham, MA, USA). The relative intensity was quantified using densitometry with ImageJ software with reference to the positive controls on the membrane.

### TGF-β1 and CXCL1 levels detection by ELISA

For the detection of CXCL1 and TGF-β1 levels in cell culture supernatants and urine samples, a standard sandwich ELISA assays were performed using the DuoSet human TGF-β1 and CXCL1 ELISA kits (R&D Systems, Minneapolis, MN, USA), according to the manufacturer’s specifications. Urinary CXCL1 and TGF-β1 were calibrated against urine creatinine before the levels from IgAN patients and healthy controls were compared.

### Total RNA extraction and RT-PCR

Total RNA was extracted from cultured cells using TRIZOL^®^ Reagent (Invitrogen, Carlsbad, CA, USA) according to the manufacturer’s instructions. Two micrograms of total RNA were reverse-transcribed to cDNA using a Reverse Transcription System (Promega, Madison, WI, USA). The cDNA was stored at -20°C until further amplification. Gene expression levels of CXCR2 and GAPDH were examined by real-time PCR using SYBR^®^ Premix Ex Taq™ (Takara, Shiga, JP) on an Applied Biosystems 7500 Real-Time PCR System. The relative mRNA expression of CXCR2 was calculated using the comparative ΔΔCt method with the following formula: relative expression of CXCR2 = 2^(-ΔΔCt), in which ΔΔCT = [(CT _CXCR2_ – CT _GAPDH_) experimental sample – (CT _CXCR2_ – CT _GAPDH_) control sample]. The primer sequences used in this study are as follows:

CXCR2 forward: GCTCTGACTACCACCCAACC;
CXCR2 reverse: AGGACACCTCCAGAAGAGCA;
GAPDH forward: AGAAGGCTGGGGCTCATTTG;
GAPDH reverse: AGGGGCCATCCACAGTCTTC.

### Western blotting

Total human podocyte extracts were separated by SDS-polyacrylamide gel electrophoresis (SDS-PAGE) and transferred onto a polyvinylidene difluoride (PVDF) membrane. After blocking, the membrane was incubated with rabbit anti-CXCR2 (1:500, Abcam, Cambridge, UK) and mouse anti-GAPDH (1:1,000; Sigma, St. Louis, MO, USA) antibodies, followed by incubation with horseradish peroxidase-conjugated secondary antibodies. Binding was detected by western lightning plus ECL reagent (PerkinElmer, Waltham, MA, USA).

### Lactate dehydrogenase assays

Lactate dehydrogenase (LDH) release into the culture medium was used to evaluate podocyte death. the CytoTox 96^®^ Non-Radioactive Cytotoxicity Assay (Promega, Madison, WI, USA) was used to detect the released LDH in podocyte supernatants, according to the manufacturer’s recommendations.

### Podocyte adhesion assay

The adhesion capacity of podocytes was determined by counting the adherent cells as previously described, with mild modifications [21–23]. Briefly, podocytes were seeded in 96-well plates and treated as previously described for 4 h. After removing non-adherent cells by washing samples with 0.1 M PBS, adherent cells were detected using the CellTiter 96 ^®^AQueous One Solution Cell Proliferation Assay System (Promega, Madison, WI, USA) following the manufacturer’s protocol. Gradient podocytes were seeded and incubated in the same manner in 96-well plates to establish a standard curve, which was subsequently used for the quantification of adherent cells.

### CXCR2 staining of urine podocytes

Immunofluorescence staining for urine podocytes was performed as previously described [24]. Briefly, a 50-ml fresh sample of the first urine in the morning was collected and centrifuged at 700 *g* for 5 min. The supernatant was carefully aspirated and the sediment pellet was then re-suspended and centrifuged onto slides. After being air-dried, the slides were fixed with acetone at -20°C for 5min, and permeabilized with 0.02% Triton^®^ X-100. After washing with 0.01 M PBS, the slides were co-incubated with mouse anti-podocylxin antibody (Abcam) and rabbit anti-CXCR2 antibody (Abcam), followed by incubation with TRITC-conjugate goat anti-mouse IgG and FITC-conjugate goat anti-rabbit IgG. Coverslips were mounted with fluoroshield mounting medium with DAPI (Abcam), which was used to stain the nuclei. The slides were imaged by a FluoView™ FV1000 confocal laser microscope (Olympus). Nucleated podocalyxin-positive cells were considered to be podocytes [25].

### Statistical analysis

Statistical analyses were performed by SPSS software (version 13.0; SPSS, Chicago, IL, USA). For Continuous variables, independent-samples t test or one-way ANOVA was used if the data was in normal distribution, and if not, Mann-Whitney U test was performed. Data with normal distribution were expressed as mean ± standard deviation, while other data were expressed as median (5% percentile and 95% percentile). A p-value of less than 0.05 was considered to be statistically significant.

## Results

### cIgA1 induced cytokine expression profile in human mesangial cells

**Figure 1:**
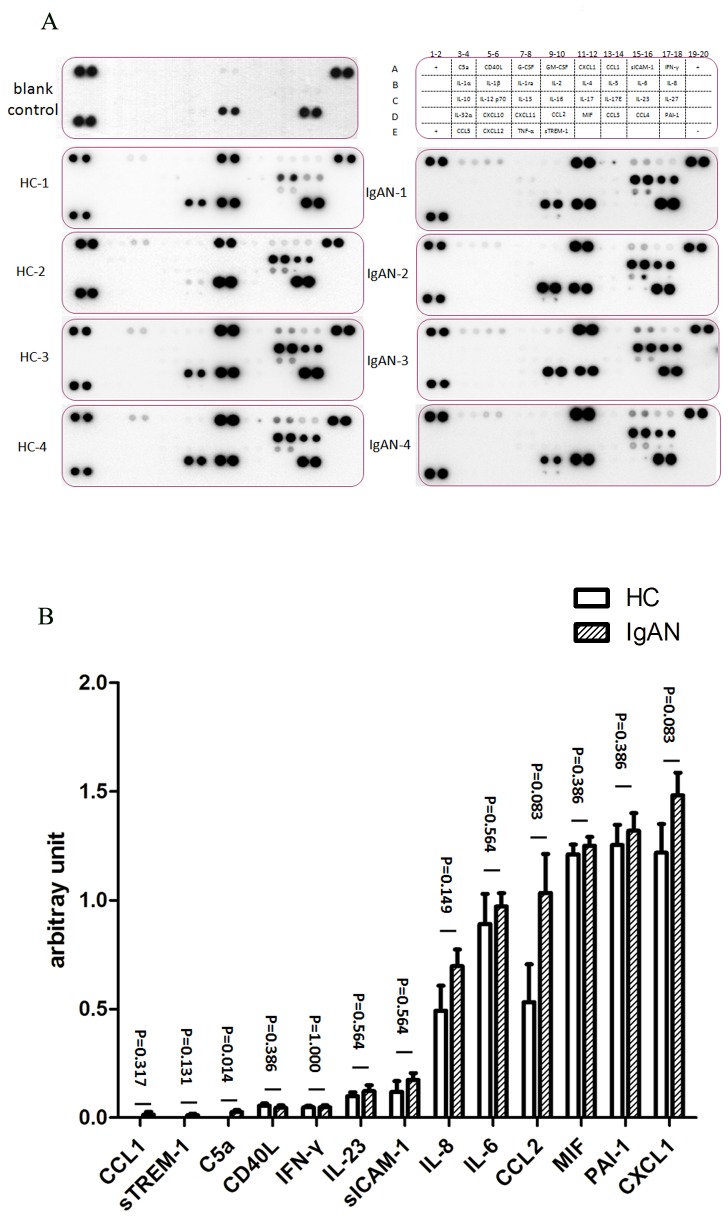
Human cytokine array analysis of multiple cytokines secreted by mesangial cells after cIgA1 treatment. A: The right upper image is a human cytokine array coordinates displaying the location of detected cytokines. Symbol + means the positive reference spot, while symbol – means the negative control. Other nine images were array results of 1ml culture medium of mesangial cells treated with 100μg/ml cIgA1 from 4 IgAN patients (IgAN-1,2,3,4), 4 health controls (HC-1,2,3,4) and without cIgA1 (blank control) for 48 hours. Images were from one minute exposures to X-ray film. B: Grouped bar chart showed multiple cytokines secreted by mesangial cells after cIgA1 treatment. Levels of CCL1, sTREM-1, C5a, IL-8, IL-6, MCP-1 and CXCL1 showed a higher tendency in mesangial medium in IgAN group than in HC group. Mann-Whitney U test was performed to compare IgAN group and HC group.

To identify cytokines released by mesangial cells under challenge of IgA1 complexes (cIgA1), a Cytokine Array was used. IgA1 complexes (cIgA1) isolated from four IgAN patients and four healthy controls were used to treat human mesangial cells (HMC). Compared with mesangial cells with non-IgA1 treatment, multiple cytokines were detected in HMC medium incubated with cIgA1 from IgAN patients or healthy controls, including MIF, Serpin E1, CXCL1, IL-6, IL-8, MCP-1, C5a, CD40L, CCL1, sICAM-1, IFN-γ, IL-23 and sTREM-1 (Figure 1A). Furthermore, the content of CCL1, sTREM-1, C5a, IL-8, IL-6, MCP-1 and CXCL1 tended to show a higher expression level in the medium of the IgAN group than in the HC group (Figure 1B). Among them, CXCL1 was found for the first time to be dysregulated by cIgA1 from IgAN patients.

### cIgA1 from IgAN patients upregulated CXCL1 and TGF-β1 in cultured human mesangial cells

**Figure 2:**
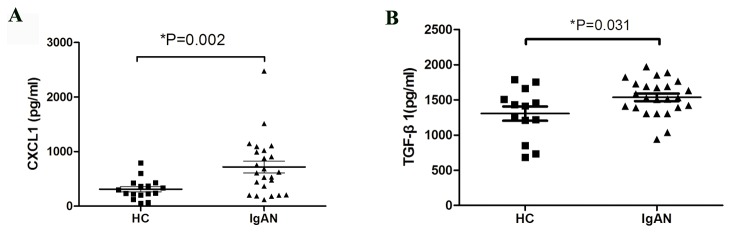
Increased secretion of CXCL1 and TGF-β1 by human mesangial cells treated with cIgA1 from IgAN patients. Compared with from healthy controls (HC), cIgA1 from IgAN patients (IgAN) significantly up-regulated mesangial cells CXCL1 (A) and TGF-β1 secretion (B) (CXCL1: 718.4 ± 532.1 pg/ml vs. 311.6 ± 191.8 pg/ml, P=0.002; TGF-β1: 1535.5±255.5 pg/ml *vs*. 1304.9±363.8 pg/ml, P=0.031).

To further validate the upregulation of CXCL1, quantitative ELISA assay was used to detect the CXCL1 levels in the HMC medium. For the validation purpose, cIgA1 from 24 IgAN patients and 16 HC were isolated and used to incubate HMC. The expression of CXCL1 by mesangial cells was significantly upregulated in the IgAN-HMC group compared to the HC-HMC group (718.4 ± 532.1 pg/ml vs. 311.6 ± 191.8 pg/ml, p = 0.002) (Figure 2A). Meanwhile, the expression of total TGF-β1 in mesangial cell medium was also detected. In accordance with previously reported results, cIgA1 from IgAN patients significantly upregulated the secretion of TGF-β1 by mesangial cells compared with that from HC (1,535.5 ± 255.5 pg/ml vs. 1,304.9 ± 363.8 pg/ml, p = 0.031) (Figure 2B). In the validation experiment, we found that cIgA1 from IgAN patients could upregulate CXCL1 and TGF-β1 in cultured human mesangial cells.

### Increased urinary CXCL1 and TGF-β1 in IgAN patients

**Figure 3:**
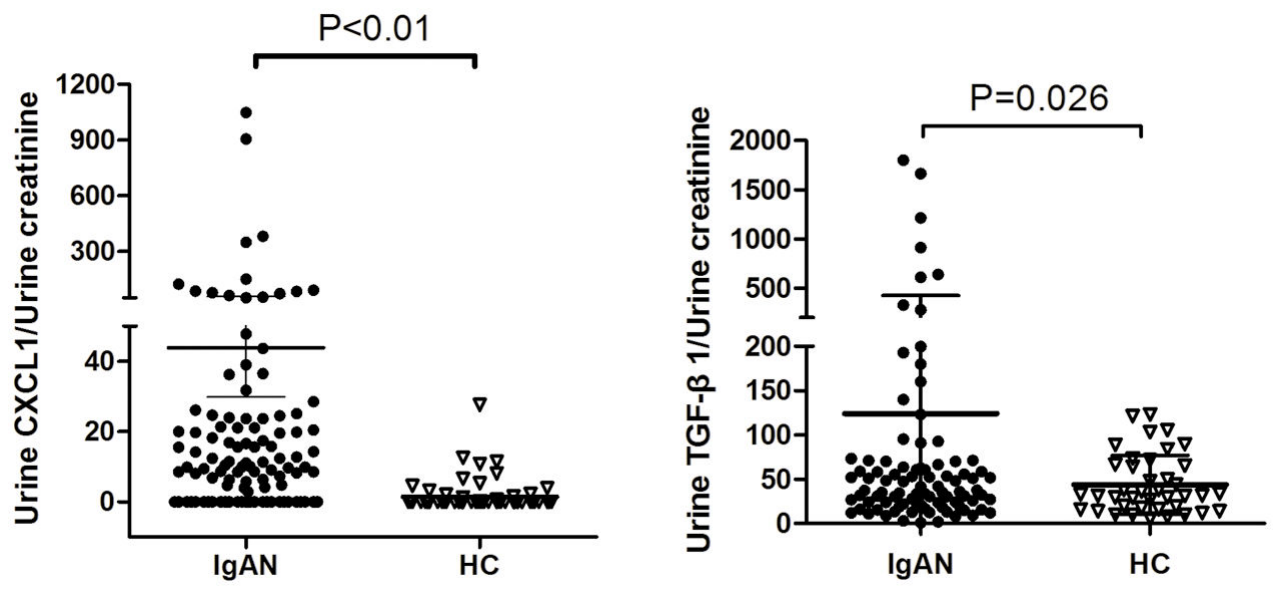
Elevated urinary levels of CXCL1 and TGF-β1 in IgAN patients. Patients with IgAN (IgAN) showed higher urinary levels of CXCL1 (A) and TGF-β1 (B) than that in healthy controls (HC) (CXCL1: 43.85±142.49 pg/mg Vs 1.38±4.09 pg/mg, p<0.01; TGF-β1: 107.56±283.95 pg/mg Vs 43.83±33.85 pg/mg, p=0.026). Individual symbols represent individual person (104 IgAN patients and 74 healthy controls).

To further evaluate the significance of CXCL1 and TGF-β1 in IgA nephropathy, we detected urinary CXCL1 and TGF-β1 in patients with IgAN. Patients with IgAN showed higher levels of urinary CXCL1 and TGF-β1 compared to HC (CXCL1: 10.71 (0.00, 144.64) pg/mg vs. 0.00 (0.00, 10.91) pg/mg, p < 0.01; TGF-β1: 37.36 (8.48, 747.85) pg/mg vs. 32.15 (7.47, 120.54) pg/mg, p = 0.026, Figure 3). Urinary TGF-β1 levels in HC were normally distributed, so we used upper 95% confidence limits (TGF-β1 = 109.99 pg/mg) as the cutoff to divide individuals into those with a higher level of urinary TGF-β1and those with a lower level. For urinary CXCL1 levels, which were not normally distributed, we created a ROC curve and chose the point with the best sensitivity and specificity (CXCL1 = 4.623 pg/mg) as the cutoff to divide individuals into those with higher or lower urinary CXCL1 levels. According to the cutoff points for these two parameters, we further classified IgAN patients into three groups: group A: lower urinary CXCL1 and TGF-β1 levels; group B: higher urinary CXCL1 and lower urinary TGF-β1 levels; and group C: higher urinary CXCL1 and TGF-β1 levels. Interestingly, we found that IgAN patients in group C had a significantly higher 24 h urinary protein excretion (UPE), a lower estimated glomerular filtration rate (eGFR) and a higher proportion of crescentic glomeruli (Table 1). The results revealed that patients with IgA nephropathy had higher urinary levels of CXCL1 and TGF-β1, and the increased urinary levels of CXCL1 and TGF-β1 were also associated with severe clinical and histological manifestations.

**Table 1:** Clinical and pathological manifestations of IgAN patients grouped regarding urinary CXCL1 and TGF-β1.

	**Group A**	**Group B**	**Group C**	**P value**
N	30	60	14	
Urine CXCL1/Urine creatinine (pg/mg)^a^	0.00 (0.00, 4.06)	15.06 (6.24, 90.77)	57.05 (4.68, 104.86)	
Urine TGF-β1/Urine creatinine (pg/mg)^a^	25.48 (0.00, 62.11)	30.53 (0.00, 90.08)	304.8 (123.24, 1801.22)	
MAP (mmHg)^b^	91.56±9.64	92.86±11.23	99. 74±13.92	0.069
UPE (g/24h)^a^	1.08 (0.32, 5.58)	1.40 (0.19, 6.23)	4.11 (0.23, 11.23)	0.001
eGFR (mL/min/1.73 m^2^)^a^	109.53±41.2	82.40±31.20	55.41±49.97	<0.001
cresentic glomeruli proportion (%)^a^	0.00 (0.00, 48.24)	6.82 (0.00, 46.15)	13.17 (0.00, 74.29)	0.018

a. Data were expressed as mean ± standard deviation.

b. Data were expressed as median (5% percentile, 95% percentile)

### TGF-β1 induced upregulation of CXCR2 in podocytes

**Figure 4:**
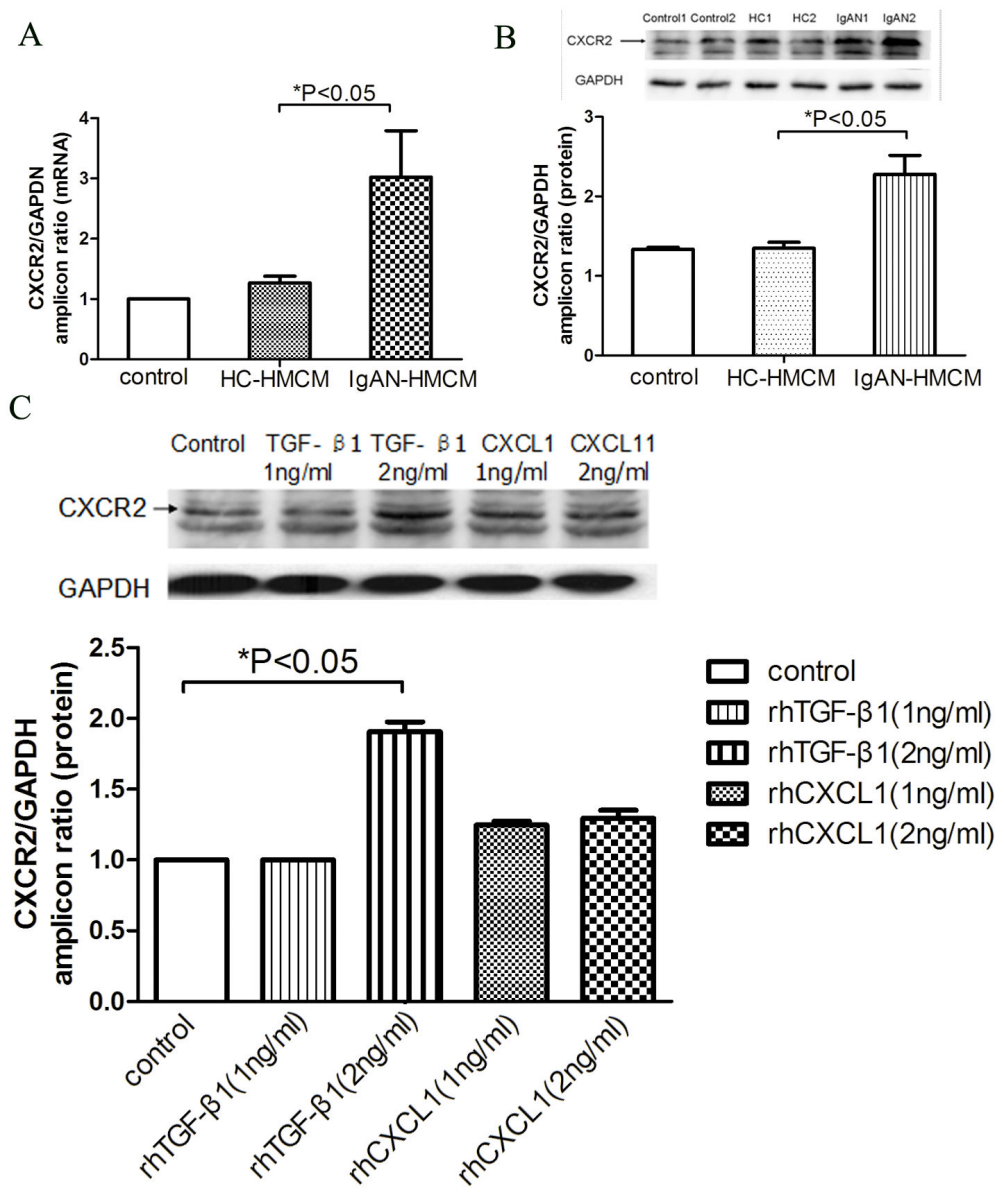
Upregulation of CXCR2 expression in podocytes cultured with conditioned medium of mesangial cells exposed to cIgA1 from IgAN patients (IgAN-HMCM) and rhTGF-β1. The mRNA (A) and protein (B) expression of CXCR2 were significantly increased in podocytes cultured with conditioned medium of mesangial cells exposed to cIgA1 from IgAN patients (IgAN-HMCM) than from healthy controls (HC-HMCM). Similar findings were observed in podocytes cultured with 2 ng/ml TGF-β1 (C), but not under rhCXCL1.

It was reported that mesangial cells secreted humoral factors could impair podocytes. To evaluate the influence of CXCL1 and TGF-β1 on podocytes, we at first investigated the expression of CXCR2, which is the receptor for CXCL1. CXCR2 was significantly upregulated in podocytes in the IgAN-HMCM group compared to the HC-HMCM group (Figure 4A and 4B). As the concentration of CXCL1 and TGF-β1 were both increased in the IgAN-HMCM group, we further investigated their respective influence on CXCR2 expression. The upregulation of CXCR2 in podocytes was induced by rhTGF-β1, but not rhCXCL1. In addition, the expression of CXCR2 in podocytes was higher under 2 ng/ml rhTGF-β1 than 1 ng/ml (Figure 4C). These findings suggested that mesangial-induced TGF-β1 upregulated expression of CXCR2 in podocytes.

### Mesangial-induced CXCL1 and TGF-β1 synergistically increased podocyte death and decreased podocyte adhesion via CXCR2

**Figure 5:**
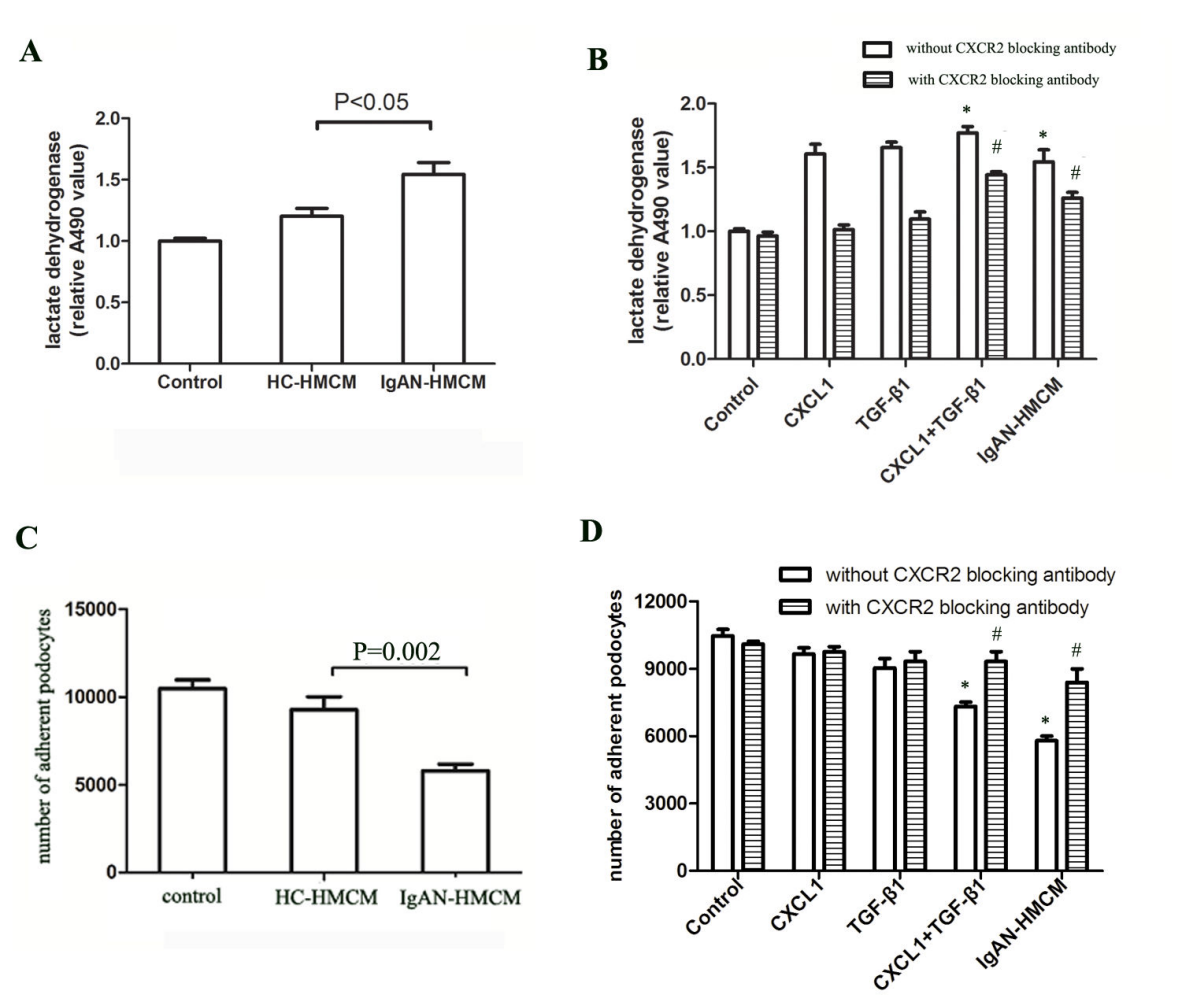
Increased podocytes death and reduced podocytes adhesion cultured with conditioned medium of mesangial cells exposed to cIgA1 from IgAN patients (IgAN-HMCM) and rhTGF-β1 together with rhCXCL1. A: The podocytes released LDH were significantly increased when podocytes were cultured with conditioned medium of mesangial cells exposed to cIgA1 from IgAN patients (IgAN-HMCM) compared to from healthy controls (HC-HMCM). B: Similar increased LDH could also be observed when podocytes were treated with 2ng/ml CXCL1 and 2ng/ml TGF-β1 together (symbol * represented p<0.05 compared with control). The increased podocytes released LDH, induced by IgAN-HMCM or rhTGF-β1 (2ng/ml) together with rhCXCL1 (2ng/ml) could be partially rescued by simultaneous incubation with 0.5ug/ml blocking antibody against CXCR2 (symbol # represented p<0.05 compared with without CXCR2 blocking antibody). C: The number of adherent podocytes were significantly decreased when podocytes were cultured with IgAN-HMCM compared to HC-HMCM (5790±377 cells *vs*. 9272±736 cells, P=0.002). D: Similar decreased podocyte adhesion could also be observed when podocytes were treated with 2ng/ml CXCL1 and 2ng/ml TGF-β1 together (7327±326 cells *vs*. 10468±499 cells, P=0.001) (symbol * represented p<0.05 compared with control). The decreased podocyte adhesion, induced by IgAN-HMCM or rhTGF-β1 (2ng/ml) together with rhCXCL1 (2ng/ml) could be partially rescued by simultaneous incubation with 0.5ug/ml blocking antibody against CXCR2 (symbol # represented p<0.05 compared to without CXCR2 blocking antibody).

Then, we investigated the effect of mesangial cells released mediators on podocyte viability. Released Lactate dehydrogenase (LDH) in podocyte culture supernatants was used as marker to evaluate podocyte death. The LDH released from podocyte incubated with IgAN-HMCM was significantly higher than with HC-HMCM (Figure 5A). Meanwhile, the number of adherent podocytes were significantly lower in the IgAN-HMCM group than in the HC-HMCM group (5,790 ± 377 cells vs 9,272 ± 736 cells, p = 0.002) (Figure 5C). Collectively, podocyte viability were decreased under IgAN-HMCM treatment.

To further explore the underlying cytokines that influence podocyte viability, rhTGF-β1 and rhCXCL1 were investigated. Compared with no cytokines, when incubated with both 2 ng/ml CXCL1 and 2 ng/ml TGF-β1, the podocyte released LDH increased significantly (Figure 5B) and the podocyte adhesion ability decreased significantly (7,327 ± 326 cells vs. 10,468 ± 499 cells, p = 0.001) (Figure 5D). Simultaneous incubation with 0.5 µg/ml blocking antibody against CXCR2 could partially rescue the podocyte damage (increased podocyte death and reduced podocyte adhesion) induced by the combined administration of 2 ng/ml CXCL1 and 2 ng/ml TGF-β1, as well as by IgAN-HMCM (Figure 5B and 5D). Thus, mesangial-induced CXCL1 and TGF-β1 synergistically increased podocyte death and decreased podocyte adhesion via CXCR2.

### Expression of CXCR2 in urine exfoliated podocytes in IgAN patients

**Figure 6:**
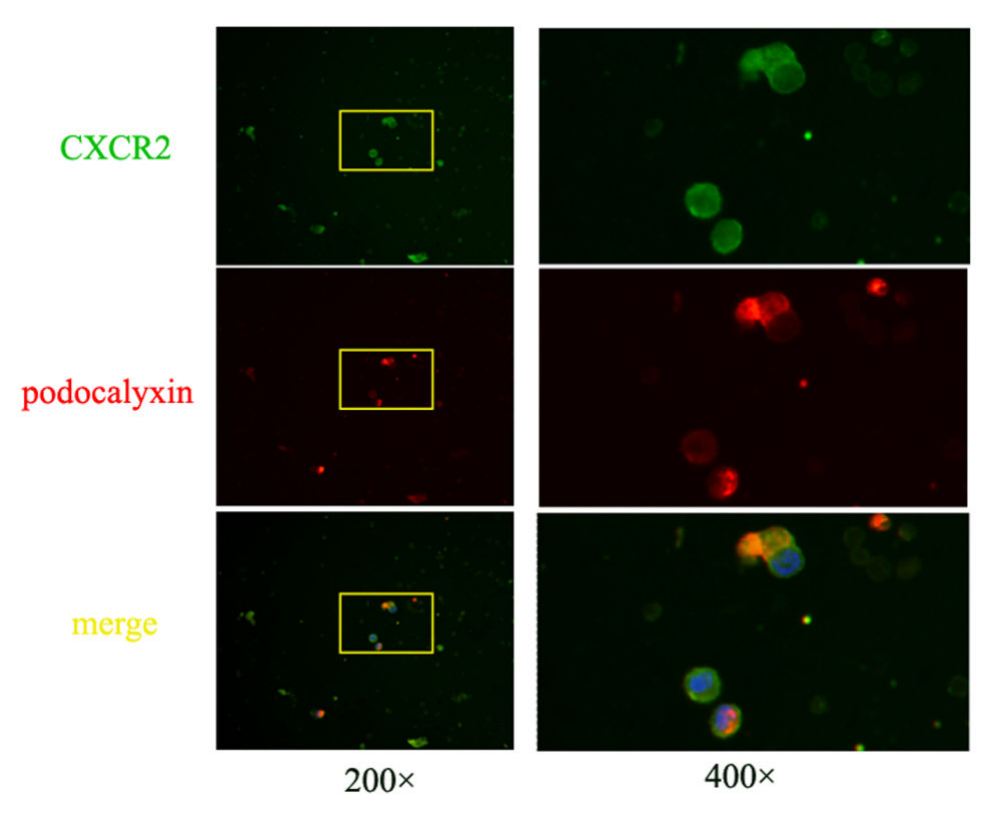
Immunofluorescence staining of CXCL1 and podocalyxin in urine exfoliated podocytes in IgAN patients. Urine exfoliated nucleated cells were stained with DAPI (blue). Expression of CXCL1 (green) staining was observed in urine exfoliated podocalyxin (red) staining positive cells. Images in left panel were showed in low magnitude (200 ×) and images in right panel were showed in high magnitude (400 ×).

To further evaluate the involvement of CXCR2 in podocyte loss in IgA nephropathy, we investigated expression of CXCR2 in urine exfoliated podocytes in 19 IgAN patients. Exfoliated podocytes, confirmed by nucleated and podocalyxin-positive cells, were found in 8 of 19 patients. Among them, 6 patients had co-expression of CXCR2 and podocalyxin in urine exfoliated cells (Figure 6). Therefore, the expression of CXCR2 on urine exfoliated podocytes supported its involvement in podocyte loss in IgA nephropathy.

## Discussion

Mesangial lesions are the most common histological findings in IgAN [1,2]. Several studies have explored the mesangial response under the challenge of IgA deposition in IgAN and revealed a series of activated mesangial phenotypes, including mesangial cell proliferation, mesangial matrix deposition and the secretion of inflammatory cytokines and chemokines, including TNF-α, TGF-β1, IL-6, IL-8, MCP-1, MIF and PAF [8,9,16,26–30]. However, most of these studies investigated only one or two cytokines rather than the complete local cytokine production profile. In the present study, a cytokine array was used to reveal the full profile of mesangial cell-derived soluble mediators after treatment with pathogenic IgA1 complexes (cIgA1). We found a tendency for increased excretion of multiple biological factors, including TGF-β1, IL-6, IL-8 and MCP-1, which were also reported previously [27,31–34], and significantly elevated CXCL1, which was a novel finding in our present study. Furthermore, higher urinary levels of CXCL1 in patients with IgAN verified its involvement in IgAN. Our findings, based on both in vitro and in vivo experiments, illustrate that mesangial cells activated by pathogenic IgA1 complex have induced CXCL1 secretion in IgAN.

The mesangial cell-derived upregulated mediator, CXCL1, has aroused substantial interest. CXCL1 is a member of the CXC chemokine family and has powerful neutrophil chemoattractant activity [35,36]. However, in IgAN, the most common infiltrating inflammatory cells are lymphocytes and monocytes, rather than neutrophils, which may result from the upregulation of MCP-1. Therefore, we hypothesized that CXCL1 may have other actions than its chemotactic function in IgAN. Very recently, Lai et al. postulated that glomerulopodocytic cross-talk existed in IgAN [7]. TNF-α, TGF-β1 and PAF were identified as mesangial-derived mediators that induce podocyte injury in IgAN [8,9,16]. Thus, our next aim was to discover whether CXCL1 acted as a mesangial-derived mediator to induce podocyte injury in IgAN.

CXCL1 is involved in a variety of pathophysiological processes, including angiogenesis, inflammation, wound healing and tumorigenesis [37,38]. CXCL1 elicits its effects by signaling through the chemokine receptor, CXCR2 [39]. It has been reported that CXCR2, the receptor for CXCL1, is expressed in the normal human glomerulus [40]. In the present study, we detected weak CXCR2 expression in cultured podocytes, which could be upregulated by rhTGF-β1, but not by rhCXCL1. The conditioned medium of mesangial cells exposed to cIgA1 from IgAN patients could also increase the expression of CXCR2 by podocytes, which at least was partly induced by elevated TGF-β1 levels in the conditioned medium, since the effect on CXCR2 of other elevated cytokines in the conditioned medium had not been investigated.

The elevated secretion of CXCL1 from mesangial cells and the upregulated expression of CXCR2 by podocytes led us to speculate that there was a synergistic effect between CXCL1 and TGF-β1 on podocytes. Our results revealed the mechanism of podocyte loss in IgAN, where pathogenic IgA1 complexes could upregulate the secretion of CXCL1 and TGF-β1 by mesangial cells which, through a glomerulopodocytic cross-talk mechanism, acted synergistically to induce increased podocyte death and impaired podocyte adhesion. Furthermore, we found that a blocking antibody against CXCR2 could partially rescue the synergistic effect of CXCL1 and TGF-β1 on podocytes, which indicated that CXCR2 was located in the common pathway for the synergistic effect of CXCL1 and TGF-β1. Meanwhile, the observation that the expression of CXCR2 by urine exfoliated podocytes in some IgAN patients reinforced the importance of CXCR2 in IgAN.

In recent years, great attention has been paid to chemokines and their receptors in acute kidney injury (AKI) and chronic kidney disease (CKD). Some researchers proposed that the inhibition of chemokines and their receptors could be a potential treatment strategy for renal disease [41]. It would indeed be a promising research direction to investigate whether CXCR2 could act as a potential therapeutic target for IgAN, and other renal diseases that have a phenotype that involves podocyte dysfunction.

In conclusion, our present study implied that the cIgA1 from IgAN patients could upregulate the secretion of CXCL1 and TGF-β1 by mesangial cells. Their synergistic effect induced the increased podocytes death and their adhesion dysfunction via CXCR2. This might be a potential mechanism for the podocyte loss observed in IgA nephropathy.
